# A finite-element approach to evaluating the size effects of complex nanostructures

**DOI:** 10.1098/rsos.160625

**Published:** 2016-12-07

**Authors:** Dingjie Lu, Yi Min Xie, Qing Li, Xiaodong Huang, Yang Fan Li, Shiwei Zhou

**Affiliations:** 1 Centre for Innovative Structures and Materials, School of Engineering, RMIT University, GPO Box 2476, Melbourne 3001, Australia; 2 XIE Archi-Structure Design (Shanghai) Co. Ltd, Shanghai 200433, People's Republic of China; 3 School of Aerospace, Mechanical and Mechatronic Engineering, The University of Sydney, Sydney, New South Wales 2006, Australia

**Keywords:** size effect, finite-element method, nanostructure

## Abstract

The size effects that reveal the dramatic changes of mechanical behaviour at nanoscales have traditionally been analysed for regular beam systems. Here, the method of using finite-element analysis is explored with the intention of evaluating the size effects for complex nanostructures. The surface elasticity theory and generalized Young–Laplace equation are integrated into a beam element to account for the size effects in classical Euler–Bernoulli and Timoshenko beam theories. Computational results match well with the theoretical predictions on the size effect for a cantilever beam and a cubic unit cell containing 24 horizontal/vertical ligaments. For a simply supported nanowire, it is found that the results are very close to the experimental data. With the assumption that nanoporous gold is composed of many randomly connected beams, for the first time, the size effect of such a complex structure is numerically determined.

## 1. Introduction

Size effects refer to the abnormal changes of mechanical properties when the characteristic sizes of nanomaterials or nanostructures are less than 100 nm [1–4]. Since the rapid increase of Young's modulus and flexural rigidity at the level of nanoscale was experimentally detected by using three-point bending tests [5,6], nano-indentation [7], atomic force microscopy [8] and Raman spectroscopy [9], size effects have attracted intense attention as they could be used in many fields like lightweight materials, sensors and actuators [10–12].

One of the most challenging problems in size effects is the establishment of an appropriate approach to evaluating the dependence of effective properties on the characteristic size. The first estimation was made by the well-known Gibson–Ashby model [13], which estimates the effective Young's modulus E* of an open-cell foam material with a density of *ρ* by E*/*E*_0_ = *C*(*ρ*/*ρ*_0_)^2^, in which the density and Young's modulus of the bulk material are represented by *ρ*_0_ and *E*_0_, respectively. Note that the constant *C* should be determined experimentally. An alternative is to use the molecular dynamics simulation to predict the mechanical properties at atomic level [14]. Considering the huge amount of particles to be included in the numerical model, this approach is computationally inefficient even for ordinary scale nanostructures [15].

Recent studies show that the strengthened or weakened mechanical properties are attributed to the strong interactions of atoms and their clusters on the material surface when the surface-to-volume ratio is extremely large at nanoscale [10,16,17]. Based on this fundamental conjecture, scientists have learned how to theoretically explain such abnormal changes using a core–shell model in continuum mechanics [10,16–18]. And they found that the main reasons for the size effects are ascribed to the surface residual tension and surface stiffness induced by the material disparity between the superficial layer and inner core [19,20].

Later, a framework of surface elasticity [19] and generalized Young–Laplace equation [20] was integrated with the beam theory to study the size effects for a ZnO nanowire and a nanostructure. The dependence of Young's modulus on the characteristic size obtained by this approach shows a similar trend to the experimental results [11,21]. The aforementioned theoretical studies are based on the models of simple beams and regular beam systems, which are theoretically viable but geometrically dissimilar to nanostructures. Later, an iterative finite-element method was proposed to directly simulate the deformation of complex nanostructures under the curvature-dependent pressure resulting from the size effects [22]. Though this computational approach can be used to validate the experimental data for randomly shaped nanoporous structures, it is inapplicable to nanostructures less than 10 nm due to the low efficiency and convergence difficulty at such a small scale.

To complement previous work, this paper aims to develop a finite-element method capable of evaluating the size effect for complex structures at any level of scale. With the assumption that the complex nanoporous structures are composed of a system of interconnected beams [23], we explicitly incorporate the size effect derived from the surface elasticity and generalized Young–Laplace equation into the element stiffness matrix. Numerical tests indicate that this method can predict the same size effect as the theoretical study in [21] for a cantilever beam and very similar data to those for a cubic unit cell containing 24 horizontal/vertical ligaments [24]. The computational results for a simply supported beam and nanoporous gold match the experimental data [25,26] within an acceptable margin of error.

## 2. Theory

The surface elasticity theory and generalized Young–Laplace equation are combined to study the size effect for a beam with circular cross section. For completeness, they are briefly reviewed before incorporating them into the Euler–Bernoulli and Timoshenko beam theories.

According to the surface elasticity theory [27], the surface stress $\tau_{\alpha \beta }^{s}$, a symmetric 2 × 2 tensor on the tangent plane to the surface, is given as

2.1 $$\tau_{\alpha \beta }^{s}=\frac{\partial G(\varepsilon_{\alpha \beta }^{s})}{\partial \varepsilon_{\alpha \beta }^{s}}+\tau_{0\_\alpha \beta }^{s}\delta_{\alpha \beta }\;(\alpha ,\beta =1,2,3),$$

where $\varepsilon_{\alpha \beta }^{s}$ is the surface strain tensor, $G(\varepsilon_{\alpha \beta }^{s})$ denotes the surface energy in the global coordinate system and *δ_αβ_* is the Kronecker delta. The initial surface stress tensor is represented by $\tau_{0\_\alpha \beta }^{s}$. By assuming that the surface is homogeneous, isotropic and linearly elastic, the overall surface stress *τ* along the longitudinal direction of a beam can be simplified to

2.2 $$\tau =\tau_{0}+E_{s}\varepsilon_{x}^{s},$$

where *E*_s_ is the effective surface stiffness and *τ*_0_ is the initial surface stress along the longitudinal direction.

According to generalized Young–Laplace equation, the stress jump Δ*σ_ij_* across the interface depends on the surface curvature *κ_αβ_* and the surface stress *τ_αβ_*, and is given as

2.3 $$\Delta \sigma_{ij}n_{i}n_{j}=\tau_{\alpha \beta }\kappa_{\alpha \beta },$$

where *n_i_* and *n_j_* denote the unit normal vectors.

**Figure 1.**
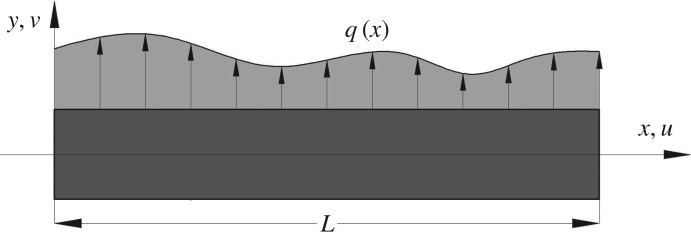
The schematic of pressure *q*(*x*) resulting from the size effects.

The surface effects with the consideration of initial surface stress can be converted into curvature-dependent pressure *q*(*x*) along the normal direction of beam surface, as shown in figure 1.

In continuum mechanics, the deformation of a beam is composed of axial displacement *u* and bending deflection *v*. Note that the introduction of size effect in traditional beam theories only influences the deflection *v* while the axial displacement remains the same. Following this assumption, the following finite-element analysis brings out a new element stiffness matrix for a two-dimensional circular cross-sectional beam. Its extension to three dimensions is straightforward if the deformations in axial and radial directions are considered.

Regarding Euler–Bernoulli beam theory, the *z*-axial displacement is neglected and the *x*-component displacement *u* is given as

2.4 $$u(x,y)=-y\frac{\partial v(x)}{\partial x}=-yv^{\prime }=-y\theta ,$$

where the deflection *v*(*x*, *y*) = *v*(*x*) only relies on *x* and the rotation angle is *θ* *=* *∂v*/*∂x = *d*v*/d*x*. The longitudinal strain is determined by differentiating the axial displacement *u*(*x*, *y*) of equation (2.4) as

2.5 $$\varepsilon =\frac{\partial u}{\partial x}=-y\frac{\partial^{2}v}{\partial x^{2}}=-y\frac{d^{2}v}{dx^{2}}=-yv^{\prime \prime }\approx -y\kappa ,$$

where *κ ≈ v^″^* denotes the curvature of the neutral axis and it can be approximated as the second derivative of deflection. Based on Hooke's law, the stress *σ* is related to ε as

2.6 $$\sigma =E\varepsilon =-Ey\frac{d^{2}v}{dx^{2}}=-Ey\kappa ,$$

where *E* is the elastic modulus. The bending moment *M* is calculated from the area integration over the cross section, given as

2.7 $$M=\int_{A}-y\sigma dA=E\kappa \int_{A}y^{2}dA=EI\kappa ,$$

where *I* denotes the moment of inertia of the cross section and *EI* is the bending rigidity.

According to the principle of virtual displacements, the total internal virtual work *δW*_I_ should be equal to the total external virtual work *δW*_E_, which is expressed as [28]

2.8 $$\delta W_{E}=\delta W_{I}.$$

The total internal virtual work *δW*_I_ of an Euler–Bernoulli beam with consideration of surface effect is given as

2.9 $$\delta W_{I}=\delta W_{\mathrm{IC}}+\delta W_{\mathrm{IS}},$$

where the subscripts ‘IC’ and ‘IS’ denote the conventional part and surface effect part of internal virtual work, respectively.

In terms of Euler–Bernoulli beam theory, item *δW*_IC_ is given by

2.10 $$\delta W_{\mathrm{IC}}=\int_{0}^{L}M_{c}\kappa dx=\int_{0}^{L}v^{\prime \prime }EIv^{\prime \prime }dx,$$

where *M*_c_ is the bending moment which is the same as the traditional definition in equation (2.7). The second term *δW*_IS_ on the right-hand side of equation (2.9) relates to the surface effect and its elaborated derivation is given below.

**Figure 2.**
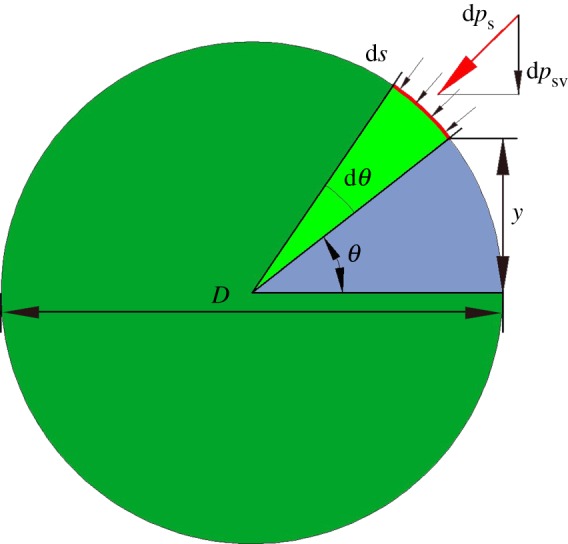
The schematic of an infinitesimal edge element along the perimeter of the cross section of a beam.

For a representative infinitesimal edge element on the perimeter of the cross section with diameter *D*, as shown in figure 2, the axial strain is [29]

2.11 $$\varepsilon_{s}=-\frac{D}{2}\sin\theta \kappa .$$

Then the surface stress along the axial direction in equation (2.2) becomes

2.12 $$\tau_{\mathrm{axial}}=\tau_{0}-E_{s}\frac{D}{2}\sin\theta \kappa .$$

In an infinitesimal arc (red curve in figure 2), this surface stress results in an extra moment about the neutral plane as

2.13 $$dM_{s}=-\tau_{\mathrm{axial}}y\;ds=\left(E_{s}\frac{D}{2}\sin\theta \kappa -\tau_{0}\right)\frac{D^{2}}{4}\sin\theta \;d\theta .$$

The overall moment of the surface effect at this cross section is obtained by integration along the perimeter of the cross section as

2.14 $$M_{s}=\int dM_{s}=\frac{\pi D^{3}}{8}E_{s}\kappa .$$

Therefore, the contribution of the surface effects to the internal work *δW*_I_ is determined as

2.15 $$\delta W_{\mathrm{IS}}=\int_{0}^{L}M_{s}\kappa \;dx=\int_{0}^{L}M_{s}v^{\prime \prime }\;dx=\int_{0}^{L}v^{\prime \prime }\frac{\pi D^{3}}{8}E_{s}v^{\prime \prime }\;dx.$$

Then, the overall internal virtual work is obtained as

2.16 $$\delta W_{I}=\delta W_{\mathrm{IC}}+\delta W_{\mathrm{IS}}=\int_{0}^{L}v^{\prime \prime }EIv^{\prime \prime }\;dx+\int_{0}^{L}v^{\prime \prime }\frac{\pi D^{3}}{8}E_{s}v^{\prime \prime }\;dx.$$

This equation indicates the beam element has an effective bending modulus of *EI* + *πE*_s_*D*^3^*/*8. Note that a similar expression was reported by He *et al.* [21].

The conventional part of the external virtual work *δW*_E_ is straightforwardly given as

2.17 $$\delta W_{\mathrm{EC}}=\int_{0}^{L}q_{c}v\;dx,$$

where *q*_c_ is the transverse force per unit length acting along the beam, and here the subscript ‘EC’ denotes the conventional part of external virtual work. With consideration of the surface effect, *δW*_ES_ can be derived similarly, where the subscript ‘ES’ denotes the surface effect part of external virtual work. According to generalized Young–Laplace equation, the axial surface stress will lead to a loading d*p*_s_ which is normal to the infinitesimal edge element (as shown in figure 2). It is expressed as

2.18 $$dp_{s}=\tau_{\mathrm{axial}}\kappa \frac{D}{2}d\theta .$$

Owing to the symmetry condition, only the component of d*p*_s_ in the flexure plane contributes to the external virtual work and it can be obtained by decomposition:

2.19 $$dp_{\mathrm{sv}}=dp_{s}\sin\theta =\tau_{\mathrm{axial}}\kappa \frac{D}{2}\sin\theta d\theta .$$

The overall surface induced transverse load can be found by integrating d*p*_sv_ along the perimeter as

2.20 $$q_{s}=\int dp_{\mathrm{sv}}ds=2\tau_{0}D\kappa .$$

Again, the expression in equation (2.20) is consistent with the distributed transverse force reported in [18,21].

Then the surface effect part of the external work *δW*_ES_ is determined as

2.21 $$\delta W_{\mathrm{ES}}=\int_{0}^{L}q_{s}vdx=\int_{0}^{L}2\tau_{0}D\kappa vdx=\int_{0}^{L}v(2\tau_{0}D)v^{\prime \prime }\;dx.$$

Therefore, the total external virtual work is computed by

2.22 $$\delta W_{E}=\delta W_{\mathrm{EC}}+\delta W_{\mathrm{ES}}=\int_{0}^{L}q_{c}vdx+\int_{0}^{L}v(2\tau_{0}D)v^{\prime \prime }\;dx.$$

In accordance with the equivalence of external virtual work *δW*_E_ and internal virtual work *δW*_I_ in equation (2.9), eventually, we use equation (2.16) and obtain

2.23 $$\int_{0}^{L}v^{\prime \prime }EIv^{\prime \prime }\;dx+\int_{0}^{L}v^{\prime \prime }\frac{\pi D^{3}}{8}E_{s}v^{\prime \prime }\;dx=\int_{0}^{L}q_{c}v\;dx+\int_{0}^{L}v(2\tau_{0}D)v^{\prime \prime }\;dx.$$

Moving the last term to the left side, this equation becomes

2.24 $$\int_{0}^{L}v^{\prime \prime }EIv^{\prime \prime }\;dx+\int_{0}^{L}v^{\prime \prime }\frac{\pi D^{3}}{8}E_{s}v^{\prime \prime }\;dx-\int_{0}^{L}v(2\tau_{0}D)v^{\prime \prime }\;dx=\int_{0}^{L}q_{c}v\;dx.$$

The expression in the above equation is similar to the weak form of the equilibrium equation in finite-element analysis. But it has two additional terms: the second term on the left side changes the effective bending rigidity while the third one comes from the extra transverse load. It is interesting to note the terms *EI* *+* *πD*^3^*E*_s_*/*8 and 2*τ*_0_*D* in equation (2.24) have been reported in pioneering studies [21] in the form of effective flexural rigidity and the coefficient resulting from the distributed transverse force.

In finite-element analysis, the elemental deflection is *v* = **Nu**^e^, in which the interpolation function **N** is a 1 × 4 vector and the nodal displacement vector **u**^e^ = [*v*_1_ *θ*_1_ *v*_2_ *θ*_2_]^T^ is a 4 × 1 vector. The second derivative of the displacement is *v*^″^ = **Bu**^e^, where **B** has a similar size to **N**. Thereby, equation (2.24) can be simplified as **K**^e^**u**^e^ = *f*^e^, in which the element stiffness matrix **K**^e^ and the element node force vector *f*^e^ are given as

2.25 $$K^{e}=\int_{0}^{L}\left(B^{T}\left(EI+\frac{\pi D^{3}}{8}E_{s}\right)B-N^{T}(2\tau_{0}D)B\right)dx$$

and

2.26 $$f^{e}=\int_{0}^{L}N^{T}q_{c}\;dx.$$

Following a similar procedure, it is straightforward to obtain the weak form for Timoshenko beam with surface effects as

2.27 $$\int_{0}^{L}EI{\left(\frac{\partial \theta_{z}}{\partial x}\right)}^{2}dx+\int_{0}^{L}\frac{\pi E_{s}D^{3}}{8}{\left(\frac{\partial \theta_{z}}{\partial x}\right)}^{2}dx+\int_{0}^{L}\psi AG{\left(\theta_{z}+\frac{\partial v}{\partial x}\right)}^{2}dx-\int_{0}^{L}2\tau_{0}D\frac{\partial \theta_{z}}{\partial x}v\;dx=\int_{0}^{L}q_{c}v\;dx,$$

where *θ_z_* is the shearing angle and *ψ* is the shear area coefficient; for solid circular cross sections *ψ* = 10/9[30].

## 3. Results and discussion

**Figure 3.**
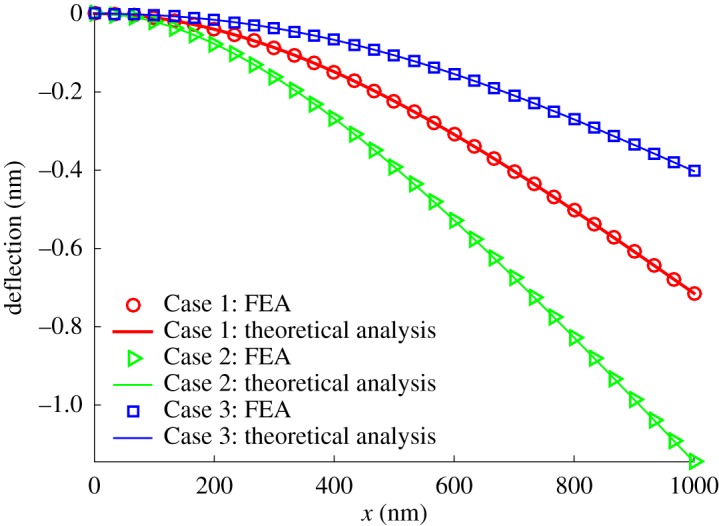
The deflections in terms of the analytical solution and finite-element analysis (FEA) for a cantilever beam.

Similar cantilever beam (fixed at the left end and loaded at the right free end with a concentrated force *p* = 0.05 nN) [21] is employed to verify this finite-element method. The beam has a length of *L* = 1000 nm, a diameter of *D* = 50 nm and a Young's modulus of *E* = 76 GPa. To investigate the roles of surface stiffness *E*_s_ and initial surface tension *τ*_0_, three cases: (1) *τ*_0_ = 0, *E*_s_ = 0, (2) *τ*_0_ = 1.22 N m^−1^ and *E*_s_ = 5.8 N m^−1^ and (3) *τ*_0_ = −1.22 N m^−1^ and *E*_s_ = 5.8 N m^−1^ are simulated and their deflection is represented by the red circular, green triangle and blue square markers, respectively, in figure 3. Encouragingly, all of them are exactly located on the solid curves which are obtained from the theoretical predictions [21]. There is no size effect in Case 1 because of *τ*_0_ = 0 and *E*_s_ = 0. Therefore, it can be used as the referent example to illustrate the significance of size effects. For example, the deflection in Case 2 (green markers) is larger than Case 1 (red markers). Thus, the beam is softened by the size effect. While the beam is strengthened in Case 3 due to the smaller deflection (blue markers).

The second example is for a clamped beam on which a concentrated load is applied at its middle point. According to Euler–Bernoulli beam theory, the deflection at beam centre is

3.1 $$v_{c}=\frac{FL^{3}}{192EI}.$$

The effective Young's modulus E* can be determined when the deflection *ν*_c_ is calculated by finite-element analysis [11,21], which is given by

3.2 $$E^{*}=\frac{FL^{3}}{192v_{c}I}.$$

**Figure 4.**
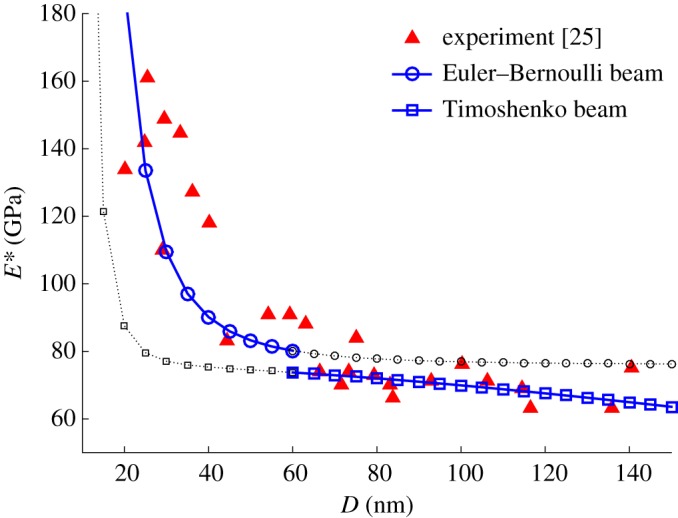
The comparison of experimental data [25] with the computational results for the effective Young's modulus of a silver nanowire.

Figure 4 demonstrates the effective Young's modulus obtained by the proposed method. According to the experiment reported in [25], the nanowires are suspended over the etched holes in the silicon wafer to form a beam with both ends fixed. In the three-point bending test [25], the length of beam is fixed to *L* = 1000 nm but the diameter *D* varies from 20 to 140 nm. According to Gere & Timoshenko [29], the ratio of diameter *D* to length *L* is *r* < 1/16 for Euler–Bernoulli beam theory and *r* > 1/16 for Timoshenko beam theory. Therefore, the threshold diameter can be determined from *L*/16 ≈ 60 nm and the valid regions of Euler–Bernoulli beam theory and Timoshenko beam theory are *D* = 0–60 nm and *D* = 60–150 nm, respectively [29]. The material of this beam is silver with *E* = 76 GPa [25], and its initial surface stress *E*_s_ = 1.22 N m^−1^ and the surface stress *τ*_0_ = 0.89 N m^−1^ are obtained from the literature [31]. As shown in figure 4, there is a sudden increase of effective Young's modulus (solid blue line with circular marker) when the beam diameter *D* < 40 nm. The numerical results match the experimental data (red triangles) within a small margin of error. Because the shear deformation is considered in Timoshenko beam theory which results in an additional energy term (the third term on the left side of equation (2.27)) in the internal energy and unchanged external energy, the effective Young's modulus becomes smaller than the one obtained from Euler–Bernoulli beam theory. Such a drop is clearly observed in figure 4 at *D* = 60 nm. The predictions beyond the validation ranges for both beam theories are plotted as black dotted lines as well, which illustrate that both theories become invalid in these ranges.

**Figure 5.**
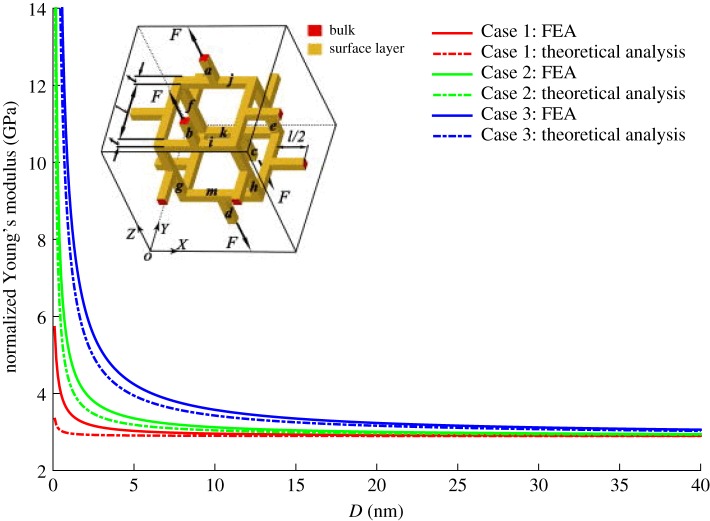
The comparison of the effective Young's modulus with the prediction in [24].

The open-cell nanoporous gold can be approximately represented by a cubic unit cell shown in the inset in figure 5. For simplicity in theoretical analysis, the constituting beams have to be perpendicular to each other. Feng *et al*. [24] indicated that the effective Young's modulus tends to abruptly increase when the beam diameter reaches 5 nm (shown as the red, green and blue dot curves for Case 1 *E*_s_ = 0.00 N m^−1^ and *τ*_0_ = 1.64 N m^−1^; Case 2 *E*_s_ = 6.60 N m^−1^; and *τ*_0_ = 1.64 N m^−1^; and Case 3 *E*_s_ = 25.00 N m^−1^ and *τ*_0_ = 1.64 N m^−1^). For this three-dimensional beam system, the effective properties (corresponding solid curves in figure 5) calculated from finite-element analysis exhibit similar trends to the dotted curves. As only the flexure deformation and the axial deformation are considered for horizontal struts and vertical struts, respectively, in [24], the system is artificially weakened and thus it is likely to reduce the stiffness. The finite-element analysis considers the system as a whole and therefore it could reveal the real deformation.

**Figure 6.**
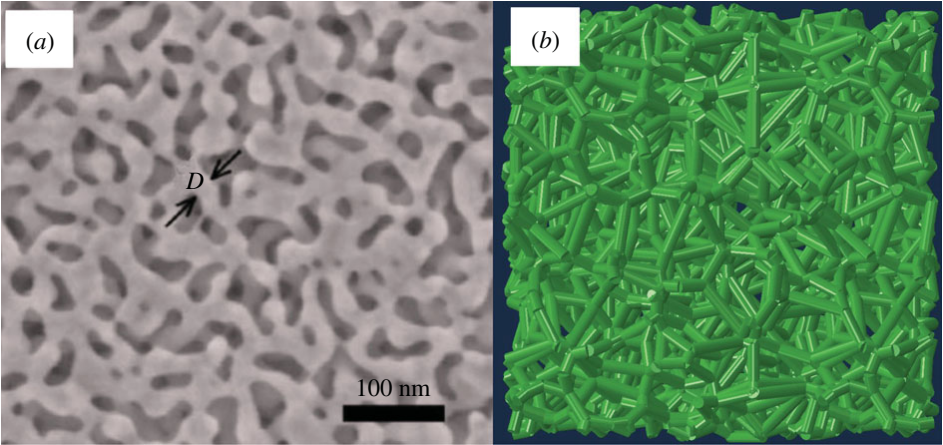
(*a*) Scanning electron microscopy image of a representative nanoporous gold with ligament size *D* = 20 nm [35]. (*b*) A perspective view of a random open-cell foam generated by Voronoi tessellations.

The most important advance of this numerical approach based on finite-element analysis is its wide applicability. It can be used for arbitrary structures in a complex shape, subjected to complicated boundary conditions, and undergoing diverse loadings as well as their combinations. Previous theoretical analyses are mainly based on simplified structures. To investigate the size effects for a real nanoporous gold as shown in figure 6*a*, we devise a cubic open-cell foam composed of some randomly connected beams (figure 6*b*) using the technique of Voronoi tessellations and Q-hull [32,33]. In this model, the diameter *D* of constituting beams ranges from 3 to 40 nm and its ratio to the length of the longest beam is fixed as *D* = 0.25*L*_max_, where *L*_max_ denotes the length of the longest beam in the model. The size of this open-cell foam is 20*D *× 20*D *× 20*D* and it has a similar volume fraction (around 0.3 in [34]) to the common nanoporous gold. Unlike previous examples based on the Euler–Bernoulli beam theory, this example is based on Timoshenko beam theory because of the relatively small ratio of the beam length to its diameter. Thereby, the element stiffness matrix for this kind of beam structure is determined from equation (2.27).

**Figure 7.**
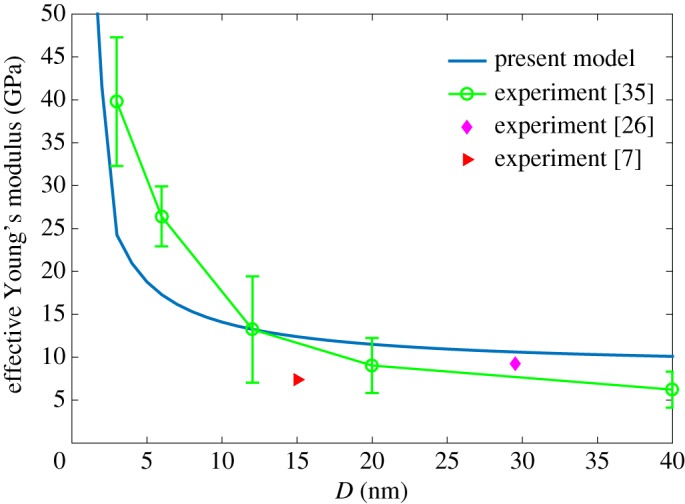
The comparison of the effective Young's modulus between computational results with experiment data [7,26,35].

For the ideal single-crystalline gold on the (001) crystal surface, the surface stress is *τ*_0_ = 1.40 N m^−1^ in accordance with the atomic simulation [31]. Given the small ligament size and post-fabrication processes may lead to a dramatic increase in residual stress, it is thereafter changed to *τ*_0_ = 80.0 N m^−1^ [26]. Young's modulus and surface stiffness for such constituting material are *E* = 79 GPa and *E*_s_ = 3.63 N m^−1^, respectively. The effective Young's modulus for this nanoporous material is plotted in figure 7 (the solid blue line), which shows it has the same trend as experiment data (green circular marker with error bar) [7,26,35]. When *D* is in the range of 15–40 nm, the effective Young's modulus is located in the 6–12 GPa range, which agrees very well with data obtained by nano-indentation and compression tests [7,26]. When *D* < 15 nm, the modulus increases rapidly and reaches 40 GPa at *D* = 3 nm. As the model generated by random spatial tessellations and Q-hull cannot fully represent the real nanoporous gold, there are disparities between the computational results and experimental data. Such errors are acceptable as they are within a marginal range. Inaccurate surface parameters might result in errors as well, but further investigations are needed in this field.

## 4. Conclusion

In summary, we develop a finite-element approach within the framework of Euler–Bernoulli and Timoshenko beam theories to retrieve the effective Young's modulus for arbitrary nanostructures. Surface effects are taken into consideration by integrating the surface elasticity theory and generalized Young–Laplace equation into the element stiffness matrix. Numerical simulation results are in good agreement with not only the analytical solution but also the experimental data. More importantly, the new elements enable the investigation of size effects for complex nanomaterials such as nanoporous gold with open-cell foams. The present approach paves the way to exploiting and optimizing the exceptional performance of material at nanoscale by using techniques such as structural topology optimization.

## Data accessibility

The paper aims to develop an algorithm to evaluate the size effect, and therefore there is no dataset.
